# Measuring subjective well-being from a multidimensional and temporal perspective: Italian adaptation of the I COPPE scale

**DOI:** 10.1186/s12955-018-0916-9

**Published:** 2018-05-08

**Authors:** Salvatore Di Martino, Immacolata Di Napoli, Ciro Esposito, Isaac Prilleltensky, Caterina Arcidiacono

**Affiliations:** 1 School of Health and Community Studies, Leeds Beckett University, Portland Building, Room 519, Leeds, LS1 3HE UK; 2 Department of Humanities, University of Naples Federico II, Via Porta di Massa 5, 80133 Naples, IT Italy; 3 School of Education and Human Development, University of Miami, Merrick Building 312, 5205 University Drive, Coral Gables, Florida 33146 USA

**Keywords:** Multidimensional well-being, Time perspective, Confirmatory factor analysis, Construct validity, Composite reliability, Measurement invariance

## Abstract

**Background:**

The objective of this study is to present the psychometric and cultural adaptation of the I COPPE scale to the Italian context. The original 21-item I COPPE was developed by Isaac Prilleltensky and colleagues to integrate a multidimensional and temporal perspective into the quantitative assessment of people’s subjective well-being. The scale comprises seven domains (Overall, Interpersonal, Community, Occupation, Psychological, Physical, and Economic well-being), which tap into past, present, and future self-appraisals of well-being.

**Methods:**

The Italian adapted version of the I COPPE scale underwent translation and backtranslation procedure. After a pilot study was conducted on a local sample of 683 university students, a national sample of 2432 Italian citizens responded to the final translated version of the I COPPE scale, 772 of whom re-completed the same survey after a period of four months. Respondents from both waves of the national sample were recruited partly through on-line social networks (i.e. Facebook, Twitter, and SurveyMonkey) and partly by university students who had been trained in Computer-Assisted Survey Information Collection.

**Results:**

Data were first screened for non-valid cases and tested for multivariate normality and missing data. The correlation matrix revealed highly significant correlation values, ranging from medium to high for nearly all congeneric variables of the I COPPE scale. Results from a series of nested and non-nested model comparisons supported the 7-factor correlated-traits model originally hypothesised, with factor loadings and inter-item reliability ranging from medium to high. In addition, they revealed that the I COPPE scale has strong internal reliability, with composite reliability always higher than .7, satisfactory construct validity, with average variance extracted nearly always higher than .5, and and full strict invariance across time.

**Conclusions:**

The Italian adaptation of the I COPPE scale presents appropriate psychometric properties in terms of both validity and reliability, and therefore can be applied to the Italian context. Some limitation and recommendations for future studies are discussed.

**Electronic supplementary material:**

The online version of this article (10.1186/s12955-018-0916-9) contains supplementary material, which is available to authorized users.

## Background

For centuries subjective well-being has been the object of philosophical investigation and in recent decades has started to attract the interest of several other disciplines, such as psychology and economics [1]. However, some have pointed out how hard – and sometimes even counterproductive – it is to find an overarching definition of this construct [2]. The scientific literature, in fact, has long acknowledged that subjective well-being has a complex and variegated nature [3, 4]. This understanding stems from at least four main theoretical traditions, that is: Hedonic, Eudaimonic, Quality of Life, and Wellness [5, 6]. Each of them offers a different and specific perspective into the meaning of subjective well-being.

According to the hedonic approach, well-being is a general experience of pleasure, satisfaction with life, absence of negative affect, and presence of positive affect [7–9]. Eudaimonic well-being entails the achievement of an optimal psychological functioning through the personal development of autonomy, environmental mastery, personal growth, positive relations with others, purpose in life, and self-acceptance [10, 11]. The quality of life (QoL) approach measures the impact of a broad range of people’s life domains, such as physical health, psychological state, level of independence, social relationships, and relationship to salient features of the environment [12, 13]. Similarly, the wellness approach considers well-being as a holistic construct including multiple areas of health and functioning, such as physical and spiritual health as well as possessing an integrated personality [14].

Based on one or a combination of the above approaches, the literature has produced a substantial number of quantitative subjective well-being instruments [14–17]. Among them, we would like to introduce to the Italian context one recently developed by Prilleltensky and colleagues [18], for the assessment of Overall, Interpersonal, Community, Occupation, Physical, Psychological, and Economic well-being (I COPPE). The theory behind the I COPPE scale posits that well-being is “*a positive state of affairs, brought about by the simultaneous and balanced satisfaction of diverse objective and subjective needs of individuals, relationships, organizations, and communities*” ([18], p. 2). This definition acknowledges that well-being is both a multilevel and multidimensional construct. It is multilevel because it emphasizes an ecological and systemic perspective that goes beyond the individual to encompass different levels of analysis. It is also multidimensional because it covers different aspects of people’s lives, which are all relevant to explain their state of well-being [6]. Moreover, this perspective brings together elements from the hedonic (satisfaction of needs), eudemonic (life fulfilment) and wellness/Quality of Life perspective (health and functioning) into an integrated tool for the subjective well-being assessment. In fact, the I COPPE scale is composed of a total of 21 items tapping into 7 correlated well-being domains (i.e. 3 items per each domain). The following is a descriptive list and operational description of the 7 domains comprising the I COPPE scale: a) Overall Well-being: positive state of affairs, as perceived by individual respondents; b) Interpersonal Well-being: satisfaction with the quality of relationships with important people such as family, friends, and colleagues c) Community Well-being: satisfaction with one’s community; d) Occupational Well-being: satisfaction with one’s job, vocation, or avocation; e) Physical Well-being: state of satisfaction with one’s overall health and wellness; f) Psychological Well-being: satisfaction with one’s emotional life; and g) Economic Well-being: satisfaction with one’s financial situation.

Beyond its multidimensional nature, a further characteristic of the I COPPE scale is the capacity to integrate a time perspective. The study of time in the evaluation of people’s well-being represents a relevant area of enquiry [19, 20]. For instance, Zimbardo and Boyd [21] have shown that a certain disposition towards time (i.e. past negative, past positive, present hedonic, present fatalistic and future) is strongly linked to different levels of well-being. In particular, past positive, present hedonic, and future predispositions are positively correlated to the experience of subjective well-being [22]. In addition, people who possess balanced time skills, that is, the capacity to shift across different time perspectives depending on the circumstances [21, 23], are generally happier than those who tend to rely only on one specific attitude to time [24].

However, time is also an element extensively overlooked – and often altogether absent – in most well-being instruments. The I COPPE scale represents a fortunate exception since it places each domain of well-being on a temporal continuum spanning from past to present and future. Its capacity to embed a temporal perspective in the assessment of subjective well-being lends this tool to several applications, among which are:

i) Comparing levels of well-being in instances where it would be impractical to retrieve information on people’s past and/or track them down in the future;
ii) Exploring the impact of life-changing events (i.e. traumas, life transitions, and turning points) and how these experiences are likely to shape people’s perception of their past, present, and future well-being.

Based on the advantages that the I COPPE scale offers, we deemed a good opportunity to introduce it to the Italian scholarship, which already boasts a well-established tradition of well-being research along with a number of already validated well-being instruments [25]. However, the latter are all limited to a selected number of life domains such as a) emotional, social, and psychological well-being, in Keyes’ Mental Health Continum Short Form [26]; b) autonomy, personal growth, environmental mastery, purpose in life, positive relations, and self-acceptance, in Ryff’s Social Scale of Well-being [27], and c) global well-being, in Diener’s Satisfaction with Life Scale (SWLS) [28]. Moreover, none of the above-mentioned instruments embeds a time perspective.

Conversely, the I COPPE scale offers the advantage of a wider range of well-being domains along with a time continuum. Therefore, we believe it represents a good addition to Italian well-being literature as well as an opportunity to bridge the gap left by the previous scales.

This tool has already been employed as part of a study comparing Italian and Serbian university students, on which occasion it showed a good level of adaptability to the Italian culture [29]. However, the authors proposed only an alternative shortened version of the I COPPE scale. This study, on the other hand, shows the results of a rigorous process of validation of its full version. In the following pages, we will report the results of construct validity, reliability, as well as model comparisons and time invariance of the Italian version of the I COPPE scale.

## Method

### Translation and back translation

The 21-item Italian version of the I COPPE scale underwent translation and back-translation [30] to establish equivalence of meaning between the source language (i.e. American English) and the target language (i.e. Italian). Following the COSMIN’s guideline [31] on cross-cultural validity, we selected four people to form the translation team (two in charge of the translations, one who oversaw the process, and the original developer). The team was composed of experienced researchers, who are proficient in both languages and who worked independently from each other during the translation and back-translation phase. Only minimal discrepancies between the two transated versions of the I COPPE were found. These were in all instances successfully resolved by the translation team.

Following Douglas and Craig’s guidelines [32], we conducted a pilot study on a local sample of university students. The pilot version of the Italian adapted I COPPE scale was completed by a local stratified random sample of 683 university students (mean age = 22.633, SD (2.827), women = 60.1%, men = 38.7%), who were at the time enrolled on Bachelor’s (73.9%) and Master’s degree (26.06%) courses at the University of Naples Federico II.

Although the scale showed psychometric good fit, χ^2^_(135)_ = 219.231, *p* < .001, CFI = .983, TLI = .974, RMSEA = .031, 90% CI [.023 .038], SRMR = .046, the qualitative oral feedback collected from the respondents suggested that we improve the readability of the questionnaire. On this account, a second phase of teamwork developed a new adaptation of the instrument, with streamlined and clearer language and instructions (e.g. the common stem question was repeated only for the first item introducing each well-being domain). This new version was presented to a small sample of informed non-specialists and further reviewed by the research team, generating a high agreement over its face validity. Therefore, we employed this as the final adapted version of the Italian I COPPE scale (available in Additional file 1).

### Samples and procedures

Following the above-mentioned changes, the final version of the I COPPE scale was completed by a national sample of 2432 Italian citizens (North = 37.1%, Centre 29.9%, and South & Islands = 32.8%). The data collected were first screened for non-Italian residents, people aged under 18 (mean age = 30.528, *SD* = 11.759), and those who did not legally consent to share their sensitive personal data or did not sign the electronic consent. The final sample consisted of 2017 respondents.

The sampling strategy made use of both convenience sampling and snowball sampling. 1291 respondents (64% of the sample) had been recruited partly trough the private contact network of the research team and in larger part through telephone interviews, which were conducted by 110 undergraduate trained students. The remaining 726 participants (36% of the sample) were recruited through online social networks (e.g. Facebook, Twitter). All participants were instructed to complete the Italian ICOPPE scale through the online SurveyMonkey platform, where they could also find information about the research and instructions on how to fill out the questionnaire.

The demographic characteristics of the sample are described in Table 1.

**Table 1:** Particiant Demographics

Local Sample (*n* = 683)		National Sample (*n* = 2017)		National Sample 2nd Wave (*n* = 696)	
Variable	Mean(*SD*)	Variable	Mean(*SD*)	Variable	Mean(*SD*)*/*
Age	22.633 (2.827)	Age	30.528 (11.759)	Age	29.412 (9.876)
Variable	Frequency in %		Frequency in %	Variable	Frequency in %
Gender		Gender		Gender	
Male	38.799	Male	40.059	Male	33.908
Female	60.175	Female	59.692	Female	66.091
Other	/	Other	0.148	Other	/
Degree Programme		Territory		Territory	
BSc Degree	73.932	North-west	24.163	North-west	20.588
MSc Degree	26.067	North-east	13.030	North-east	6.029
Faculty		Centre	29.955	Centre	28.823
Psychology	18.448	South	26.010	South	37.500
Law	15.373	Islands	6.839	Islands	7.058
Biology	14.348	Civil Status		Civil Status	
Politics	15.226	Single	34.754	Single	34.054
Engineering	15.666	With partner	41.249	With partner	46.320
Medicine	14.787	Married	20.079	Married	16.305
Other	6.149	Separated	1.388	Separated	1.443
Curriculum		Divorced	0.991	Divorced	1.010
Humanities	51.830	Widowed	0.446	Widowed	.432
Sciences	48.169	Other	1.090	Other	.432
		Education		Education	
		Primary School	.594	Primary School	.431
		Middle School	8.279	Middle School	4.885
		High School	51.313	High School	42.959
		Univ. Degree	29.846	Univ. degree	40.660
		PhD/Doctorate Specialization	8.824	PhD/Doctorate Specialization	10.632
		Other	1.140	Other	.431
		Occupation (Sector)		Occupation (Sector)	
		Managerial/Professional	11.695	Managerial/Professional	12.835
		Employee	26.251	Employee	23.582
		Secondary sector	.919	Secondary sector	.298
		Third sector	7.201	Third sector	7.462
		Student	39.968	Student	41.940
		Other	14.964	Other	13.880
		Occupation (Status)		Occupation (Status)	
		Unemployed	25.582	Unemployed	29.106
		Full-time	35.944	Full-time	35.158
		Part-time	11.601	Part-time	12.103
		Retired	1.735	Retired	1.152
		Other	24.838	Other	22.478

The undergraduate students that collaborated in the recruitment phase, were appropriately trained during a 7-day workshop in telephone interview condution and Computer-Assisted Survey Information Collection (CASIC) [33, 34]. During the recruitment phase, they used Prepared Data Entry (PDE) to direct the respondents to the online survey. They resorted to Computer Assisted Telephone Interview (CATI) only whenever necessary, to help those in need of assistance (e.g. no access to the Internet or lack of IT skills, the visually-impaired, and some older people) to fill out the online questionnaire.

Data were transferred to Mplus 7.0 and checked for possible biases due to the employment of the two methods of data collection. Wald test showed that there was no statistically significant difference at the chosen 5% alpha level between participants contacted by students and those recruited exclusively through online social networks, W_(21)_ = 24.551, *p* = .267.

A total of 1443 respondents from the national sample (71.5% of the sample), had given their availability to be re-contacted in the future for a second administration of the I COPPE scale.

The second wave was launched 4 months after the end of the first wave, through the previously employed on-line social networks along with email invites sent via the SurveyMonkey platform.^1^ After 322 individuals had answered our survey, a new group of 17 trained undergraduate students re-contacted the rest of the participants, recruiting a further 450 people. Of these, 76 were excluded due to lack of information necessary to match them to their previous data. The final sample comprises a total of 696 respondents. Once more, Wald test shows no statistically significant difference between participants contacted by the students and those who answered our mail invites, W_(21)_ = 32.132, *p* = .056. At the end of each wave, we awarded a raffle prize to a randomly selected respondent. The prizes consisted of €100 and €200 Amazon vouchers for the first and second wave respectively, which was intended as a way of thanking the respondents for their participation.

## Results

### Data analysis

Based on the results of the original validation, we used Confirmatory Factor Analysis as implemented in Mplus 7.0, to assess the applicability of the I COPPE scale to the Italian context.^2^ The correlation matrix (see Additional file 2) reveals that all the manifest variables used for the I COPPE scale are significantly correlated at the 1% alpha level. In addition, all congeneric variables show medium to high correlations, except for OV_WB_PA and OV_WB_FU, *r* = .274, *p* = .001 (see Additional file 2). Mardia’s test [35] revealed a clear violation of multivariate normality for both skewness (M = 5.386, *SD* = 0.186, *p* < .001) and kurtosis (M = 482.585, *SD* = 1.415, *p* < .001). To address this issue, Maximum Likelihood Robust (MLR) was chosen as main estimator.^3^

To assess model fit, we followed Hu and Bentler’s guidelines [36] according to which the Comparative Fit Index (CFI) and Tucker–Lewis Index (TLI) should be > .95, the Root Mean Square Error of Approximation (RMSEA) < .05, and the Standardized Root Mean Square Residual (SRMR) < .08. In addition to these, the Chi-square value should not be significant at the 5% alpha level. However, the sensitivity of this test to sample size has been highlighted on several occasions [37, 38] and since the samples recruited in this study are all relatively large, we will ignore its statistical significance.

Missing values were in all instances treated with listwise deletion, with a relatively small loss of cases in all instances. Nonetheless, power analysis based on the RMSEA test of close fit shows that at the 5% alpha level, with 118 degrees of freedom,^4^ the minimum sample size to reach a power of .8 is 117.968. This shows that the main analyses we carried out on the Italia I COPPE scale have enough power to confidently avoiding making a Type II error.

As in the original validation of the I COPPE, we allowed residual errors to correlate between manifest variables that shared an item stem, given the hypothesised method effect by time period referenced [39]. Being that this case is a Nonstandard Confirmatory Factor Analysis Model with correlated errors, we applied the rules suggested by Kenny, Kashy, and Bolger ([40], p. 253–254) for identifying our model, that is:

- each factor has at least three indicators whose errors are uncorrelated with each other,
- for every pair of constructs there are at least two indicators, one from each construct, that do not have correlated measurement error between them, and
- for every indicator, there must be at least one other indicator (not necessarily of the same construct) with which it does not share correlated measurement error.

**Fig. 1:**
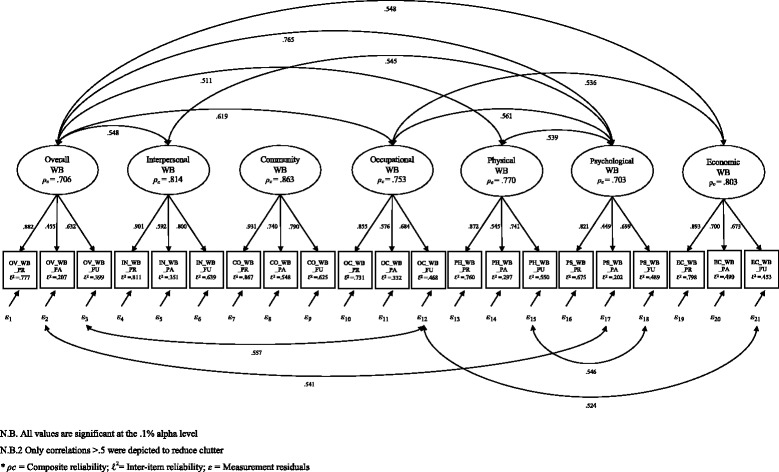
Italian I COPPE 7-factor correlated-traits model

Since all the above conditions are satisfied (see Fig. 1 and Additional file 2), we can consider the proposed Nonstandard Confirmatory Factor Analysis Model as identified.

### Results

The proposed 7-factor correlated-traits model provides a very good fit to the data, χ^2^_(118)_ = 155.413, *p* = .011, CFI = .997, TLI = .995, RMSEA = .013, 90% CI [.006 .018], SRMR = .024, therefore we can accept the null hypothesis that the model’s implied variance-covariance matrix [*Σ*(θ)] and the model’s covariance matrix [*Σ*] are not significantly different.

Figure 1 shows that nearly all congeneric variables have both significant and high factor loading associated to their corresponding latent variable. However, it is also worthy of notice that all the items of past well-being have generally lower standardised factor loadings (λ) and inter-item reliability (R^2^) than their congeneric variables. Among them, two manifest variables show the lowest values, namely OV_WB_PA, λ^0^ = .455, SE = .022, 95% CI [.412, .499], R^2^ = .207 and PS_WB_PA, λ^0^ = .449, SE = .025, 95% CI [.401, .498], R^2^ = .202.

Although some suggest that standardized loading estimates should be ideally ≥ .5 for CFA [41], none of them is small enough (i.e. <.40) not to be considered a “salient” factor loading [42].

### Model comparisons

Some studies have demonstrated that alternative structures of the I COPPE scale such as the One-Factor [29] and Bi-Factor [43] solution can better express the variability of subjective well-being in different cultural contexts. On this account, we decided to compare the 7-factor correlated-traits model proposed as the original I COPPE scale (Model A) to a series of alternative nested models (see Table 2) to test which one of these would be the most appropriate to apply to the Italian context.

**Table 2:** Model Comparisons between the 7-factor correlated-traits model and alternative models

Model/Indices	A 7 Factors	B 2nd Order	C Multi-Trait Multi-Method^b^	D Bi-Factor^c^	E One Factor
MLR χ^2^	155.413	255.003	344.343	149.167	345.788
χ^2^ df	118	132	146	108	121
χ^2^ *p*	.011	<.001	<.001	.0054	<.001
CFI	.997	.991	.986	.997	.984
TLI	.995	.986	.979	.994	.972
RMSEA (90% CI)	.013 (.006 .018)	.022 (.018 .026)	.026 (.023 .030)	.014 (.008 .019)	.031 (.027 .035)
SRMR	.024	.032	.025	.022	.028
Akaike (AIC)	147,929.900	148,049.431	147,888.189	147,938.931	148,205.576
Bayesian (BIC)	148,678.395	148,719.725	148,480.121	148,743.285	148,937.314
Model Comparison	/	B Versus A	C Versus A	A Versus D	E Versus D
∆MLR χ^2a^	/	95.685	179.258	6.882	175.441
∆df	/	14	28	10	13
∆*p*	/	<.001	<.001	.737	<.001

^a^Corrected Values; ^b^OV_WB_PR, CO_WB_PR, PS_WB_PR loading on only their method factor; ^c^OV_WB factor loading on only the general factor

Given the presence of multivariate non-normality, MLR was used as an estimator in all cases. As such, model comparisons were based on the scaled Chi-square difference statistic [44].

In the Second Order solution (Model B), the 7 factors measured in Model A were additionally constrained to load onto a higher order well-being factor. In the MTMM solution (Model C), in addition to Model A, we constrained all the items of the past, present, and future to load on a Past, Present, and Future trait factor respectively in a correlated trait-correlated method model (CTCM). The Bi-Factor solution (Model D) includes a general factor that is orthogonal to the 7 specific factors proposed in Model A. Compared to the previous solutions, Model A is nested within Model D, therefore a significant Chi-square difference statistic would favour the Bi-Factor solution and vice versa. Consistent with what was found by Myers and colleagues [45], the manifest variables of OV_WB loaded significantly only onto the general factor. Lastly, in the One Factor solution (Model E) all the 21 items comprising the I COPPE were allowed to load onto only one general well-being factor. Since Model E is not nested within Model A, it was not possible to compare them through the scaled Chi-square difference statistic. However, the Akaike and (AIC) and Bayesian (BIC) indices displayed in Table 2 – which are useful to compare non-nested models – show that Model A yields a better fit to the data than Model E. In addition, both models are nested within Model D, therefore we could make an indirect comparison, first between Model E and Model D, which favours model D, and then between Model A and Model D, which in turn favours Model A.

Since none of the alternative models proposed provides a better fit to the data than the comparative model (see Table 2), we can conclude that the multidimensional solution with 7 intercorrelated well-being factors is the best fitting model to describe the Italian adaptation of the I COPPE scale.

### Reliability and construct validity

To test for the internal reliability of the Italian I COPPE scale, we used Composite Reliability (CR), which is known to perform better than the most commonly-used Cronbach alpha, given the multitude of cases where the condition of tau-equivalence cannot be met [46]. Values of ρ_c_ > .6 are considered desirable, and above .7 are indicative of a high level of CR. Table 3 shows that *ρ*_*c*_ ranges from a minimum of .703 to a maximum of .863, indicating a good level of reliability per each factor of the I COPPE scale.

**Table 3:** Factor Correlations, Reliability and Validity Measures of the Italian I COPPE scale

Latent Variable	OV_WB	IN_WB	CO_WB	OC_WB	PH_WB	PS_WB	EC_WB
OV_WB	1						
IN_WB	.548	1					
CO_WB	.439	.299	1				
OC_WB	.619	.343	.418	1			
PH_WB	.511	.424	.323	.389	1		
PS_WB	.765	.545	.410	.561	.539	1	
EC_WB	.498	.295	.357	.536	.422	.466	1
Reliability and Validity Measures							
CR (*ρ*_*c*_)	.706	.814	.863	.753	.770	.703	.803
AVE (${\rho}_{\overline{v}}$)	.461	.601	.679	.510	.535	.455	.580
MSV	.585	.300	.192	.383	.314	.585	.287
ASV	.328	.178	.150	.219	.194	.312	.190

N.B. All values are significant at the .1% alpha level; *CR* Composite Reliability, *AVE* Average Variance Extracted, *MSV* Maximum Squared Shared Variance, *ASV* Average Shared Square Variance

Evidence of the I COPPE’s construct validity has been so far ested through Campbell and Fiske’s [47] Multitrait-Multimethod Matrix (MTMM) (see [18, 43]). However, the latter has been strongly brought into question, above of all for leaving to the researcher’s interpretation the maginitude of the correlations within the MTMM matrix [48]. For this reason, we opted for another widely used alternative, namely the Fornell and Larcker’ method (1981), which offers clearer guidelines and cut-off points to assess convergent and discriminant validity (see [41]). Indeed, according to Fornell- Larcker, convergent validity can be assessed through Average Variance Extracted (AVE), which measures the total amount of variance of a construct compared to the variance due to measurement error. According to Fornell and Larcker, values of ${\rho}_{\overline{v}}$above .5 are index of desirable AVE. In addition, AVE can also be used to test for discriminant validity. In this case, AVE should be higher than Maximum Shared Variance (MSV), and Average Shared Variance (ASV). As we can see from Table 3, all the latent variables of the I COPPE scale meet the above criteria, except for OV_WB (MSV = .585 > ${\rho}_{\overline{v}}$= .461) and PS_WB (MSV = .585 > ${\rho}_{\overline{v}}$= .455). This is probably due to the high correlation between these two factors ψ_(OV_WB, PS_WB)_ = .765 (see Table 3 and Fig. 1), the low parameter estimates for OV_WB_PA, λ^0^ = .455, *p* < .001, 95% CI [.412, .499] and PS_WB_PA, λ^0^ = .449, *p* < .001, 95% CI [.401, 498], and the significant high zero-order correlation between their error terms, ***ε*** = .541, *p* < .001. However, this does not pose a serious threat either to their convergent validity (the AVE of both factors is only slightly below the suggested cut-off point ) or to their discriminant validity (the AVE of both factors is always higher than their corresponding ASV).

### Time invariance

In this paragraph we will test the factor equivalence of the I COPPE scale across time (i.e. first and second wave). A commonly adopted practice to test for time invariance in Structural Equation Modeling (SEM) is to start with a group-specific baseline model whereby a partial measurement invariance [49, 50] is compared against increasingly constrained SEM models. The first level of invariance tests for equivalence of factor loadings (metric invariance). The next level builds upon metric invariance to further test for equivalence of intercepts (scalar invariance) [51]. Although we also provided evidence of factor invariance and equivalence of indicator residual variances (strict invariance), some argue that the latter might be unduly restrictive and that achieving partial scalar invariance is sufficient in many circumstance [36, 42, 52]. In the light of this, we tested increasingly more restrictive invariances until model fit criteria indicated that the latest set of restrictions is no longer tenable for the data, as recommended by Geiser ([53], p. 101).

To test for the time invariance of the Italian I COPPE scale, we compared the data of 696 respondents, who took part in both the first and second wave of the national sample. The time range of responses that were provided, varies between a minimum of four and a maximum of seven months from the last administration of the scale.

The presence of missing data between the two waves required deleting 118 cases, reducing the final sample to 578 cases. However, this did not significantly alter the power of the test, which still shows a 99% chance of correctly accepting the null hypothesis that the increasingly restricted models are not significantly different from the configural model.

The configural Model 1.1 provides a satisfactory fit to the data, χ^2^_(629)_ = 1005.602, *p* < .001, CFI = .966, TLI = .954, RMSEA = .032, 90% CI [.028, .036], SRMR = .066.

Comparisons between Model 1.2 versus Model 1.1 (Full Metric Invariance), Model 1.3 versus Model 1.2 (Full Scalar Invariance), and Model 1.4 versus Model 1.3 (Full Strict Invariance), show that factor loadings, intercepts, factor variance, and indicators residual variance, all are equivalent across the two-time points considered (Table 4). Therefore, we can conclude that Full Strict Invariance holds for the Italian I COPPE scale.

**Table 4:** Italian I COPPE scale Time invariance results (1st and 2nd wave)

Model/Indices	1.1 Configural Model	1.2 Full Metric Invariance	1.3 Full Scalar Invariance	1.4 Full Strict Invariance
MLR χ^2^	1005.602	1021.211	1040.046	1055.228
χ^2^ df	629	643	658	686
χ^2^ p	<.001	<.001	<.001	<.001
CFI	.966	.966	.966	.968
TLI	.954	.955	.955	.960
RMSEA (90% CI)	.032 (.028 .036)	.032 (.028 .036)	.032 (.028 .035)	.030 (.026 .033)
SRMR	.066	.067	.067	.070
Model Comparison	/	1.2 Vs 1.1	1.3 Vs 1.2	1.4 Vs 1.3
∆MLR χ^2a^	/	17.677	14.561	24.942
∆df	/	14	15	28
*P*	/	.222	.483	.631

^a^ Corrected Values

## Discussion

The findings presented in this study confirm the original structure identified by Prilleltensky and colleagues [18], since all the alternative models proposed fail to describe the data better than the 7-factor correlated model. This indicates that we can consider the Italian adapted I COPPE scale as a multidimensional instrument tapping into different, yet related, domains of subjective well-being.

The value of CR showed that all the factors of the I COPPE scale have a high level of internal reliability. Furthermore, AVE provided strong evidence of both convergent and discriminant validity, except for Overall Well-being and Psychological Well-being. Although their validity is still partially tenable, we could suggest at least two strategies for future researchers, should they encounter a more severe lack of validity [54]. The first is to delete the items with the largest measurement error variance (i.e. Overall and Psychological Past Well-being). In our case, the model fit remains nearly unaltered, χ^2^_(92)_ = 124.992, *p* = .012, CFI = .997, TLI = .995, RMSEA = .013 (.007, .019), SRMR = .022, with a substantial increase in AVE for both Overall Well-being, ${\rho}_{\overline{v}}$ = .586 and Psychological Well-being ${\rho}_{\overline{v}}$ = .586, which are now only slightly smaller than their corresponding Maximum Shared Variance = .594. Another strategy would be to collapse the overlapping dimensions into a single factor. Although the resulting model fit is statistically equivalent to the correlated seven-factor model due to the lack of over-identification, the second-order factor shows a very good level of AVE, ${\rho}_{\overline{v}}$ = .768, which is much higher than its corresponding MSV = .458.

The test for time invariance shows that the Italian I COPPE scale is consistent in measuring the 7 domains of subjective well-being across time. This is in line with similar results found in the literature, which suggest that subjective well-being might be a stable psychological trait [55, 56], in that it is unlikely to be permanently influenced by the respondent’s situational variability such as daily mood, and more likely to be affected by life-changing events and/or contextual variables [45, 57].

Lastly, all the manifest variables used show a strong relation to their corresponding domain of subjective well-being, except for the items of the past. Our findings are consistent with some previous analyses conducted on the I COPPE scale [45], which concluded that “*an individual’s perceptions of the past, at least in some circumstances, may offer negligible empirical contributions over and above an individual’s perceptions of the present and future in the practical assessment of multidimensional well-being*” (p. 796).

### Limitations

The generability of the results presented in this paper should be interpreted in the context of some limitations. Although we strived not to pose restrictions to the participation in this study, a high number of respondents who took the online survey had to be contacted through snowball sampling and convenience sampling strategies. In addition, the majority of respondents had a level of IT literacy and access to a computer, the internet, and social networks and even the small percentage who were assisted through CATI still owned a telephone. This poses some limitations to the generalisability of the results to the whole of the Italian population. Future uses of the I COPPE scale with random national samples could offer further evidence to the results we obtained.

Another limitation pertains to the number of well-being domains composing the I COPPE scale. In the original validation study, Prilleltensky and colleagues [18] identified a possible limitation of the I COPPE scale in “*the possibility that other potentially important factors… also contribute meaningfully to well-being*” (p. 212–213). In that regard, Linton and colleagues [14] recently conducted a systematic review on self-reported measures of well-being, in which they showed that the I COPPE scale encompasses six of the seven domains they found to be core components of subjective well-being, that is: overall well-being, mental well-being, social well-being, physical well-being, spiritual well-being, activities and functioning, and personal circumstances. We suggest that a future revised version of the I COPPE scale integrates Spiritual Well-being, the only relevant domain currently missing. This would contribute to placing this tool among the most comprehensive quantitative instruments for the assessment of subjective well-being.

We should also bear in mind that the I COPPE scale was designed primarily to measure subjective well-being at the individual level of analysis. As such, it should always be used in combination with other objective indicators as well as further methods to assess well-being at the community and social level [58–60].

Lastly, the I COPPE scale remains a quantitative instrument for the general assessment of people’s multiple domains of subjective well-being. Therefore, its use should be discouraged – or at least readapted – in contexts where specific life circumstances play a strong role in people’s assessment of their own subjective well-being.

## Conclusions

Our results provide empirical evidence in support of the thesis that the Italian adaptation of the I COPPE scale is a valid and reliable instrument. Indeed, evidence of good construct validity (i.e. convergent and discriminant validity), coupled with strong internal reliability and time invariance support our thesis that the I COPPE scale can be adapted to the Italian context. However, given the nature of the sampling strategies we used, we still advice caution in generalising the results presented here to the whole of the Italian population.

The main strength of this tool lies in its multidimensional nature, which encompasses nearly all the key components of subjective well-being currently identified in the literature. In addition, the I COPPE scale is almost unique in incorporating time variability, showing how people’s perception of their subjective well-being is likely to change from past to present and future.

Therefore, we believe that the evidence offered in this study constitutes an opportunity for Italian scholars, clinicians, activists, and practitioners to further investigate the nature of subjective well-being from a multidimensional and temporal perspective. In that regard, this tool can contribute to expand those research fields such as community psychology, public health, and health economics, only to name a few, that are currently investiganting – both in Italy and abroad – the intrinsic relationship between well-being and the resources provided by the environment. The flexibility in its use at the individual, organizational, and community level, makes the I COPPE scale a window onto the contextual nature of subjective well-being while acknowledging its strong link with multiple domains of life and temporal variability.

## Additional files

Additional file 1: I COPPE Scale Italian Adaptation. (DOCX 337 kb)

Additional file 2: Manifest variables Correlation Matrix. (DOCX 18 kb)

## Abbreviations

ASV: Average Shared Variance
AVE: Average Variance Extracted
CFI: Comparative Fit Index
CI: Confidence Interval
CO_WB: Community Well-being
CR: Composite Reliability
EC_WB: Economic Well-being
FU: Future
IN_WB: Interpersonal Well-being
MLR: Maximum Likelihood Robust
MSV: Maximum Shared Variance
OC: Occupational Well-being
OV_WB: Overall Well-being
PA: Past
PH_WB: Physical Well-being
PR: Present
PS_WB: Psychological Well-being
R^2^: Individual Inter-item reliability
RMSEA: Root Mean Square Error of Approximation
SD: Standard Deviation
SE: Standard error
SEM: Structural Equation Modeling
SRMR: Standardized Root Mean Square Residual
TLI: Tucker–Lewis Index

## Footnotes

1 In the absence of clear guidelines in the literature, we opted for a time interval we believed to be sufficiently wide to avoid recollection biases.

2 All the analyses and results described in this and the next paragraphs refer to the national sample

3 All the congeneric variables composing the I COPPE are measured on a ratio scale ranging from 0 to 10.

4 These figures pertain to the 7-factors correlated-traits model, which was used as main explanatory model.
