# Supplementary material for: Measuring subjective well-being from a multidimensional and temporal perspective: Italian adaptation of the I COPPE scale
Source: Health Qual Life Outcomes. 2018 May 8;16:88. doi: 10.1186/s12955-018-0916-9 (PMC5941326; doi:10.1186/s12955-018-0916-9)
Supplement: Supplementary file 1 — I COPPE Scale Italian Adaptation. (DOCX 337 kb) [file 12955_2018_916_MOESM1_ESM.docx]

**Additional Files**

**Additional file 1: I COPPE Scale Italian Adaptation**

**Recommended introduction to the survey:**

Nelle pagine successive ti verranno chieste delle domande su alcuni aspetti della tua vita. Ti

preghiamo di rispondere con sincerità a ciascuna di esse.

Non ci sono limiti di tempo, tuttavia ti consigliamo di non valutare troppo a lungo le tue risposte; ricordati che, di solito, la prima reazione è anche la più accurata.

Ti ricordiamo, inoltre, che non ci sono risposte giuste o sbagliate a questo questionario; ognuno valuta la propria vita in maniera diversa dagli altri, per cui sentiti libero/a di dare la risposta che ritieni più adatta per descrivere la tua condizione.

Ci auguriamo che il questionario sia di tuo interesse.

Grazie mille per la tua collaborazione.

**Overall Well-Being (OV_WB): positive state of affairs, as perceived by individual respondents.**

**PR:** Sulla scala orizzontale, il numero che si trova più a destra, dieci (10), rappresenta il meglio, il numero più a sinistra, zero (0), rappresenta il peggio. Considerando com’è la tua vita in questo periodo, quale numero sceglieresti?

(peggio)

0

1

2

3

4

5

6

7

8

9

(meglio)

10

**PA:** Considerando com’era la tua vita un anno fa, quale numero sceglieresti?

(peggio)

0

1

2

3

4

5

6

7

8

9

(meglio)

10

**FU:** Considerando come potrà essere la tua vita tra un anno, quale numero sceglieresti?

(peggio)

0

1

2

3

4

5

6

7

8

9

(meglio)

10

**Interpersonal Well-Being (IN_WB): satisfaction with the quality of relationships with important people such as family, friends, and colleagues.**

**PR:** Questo gruppo di domande riguarda le tue relazioni personali. Il numero che si trova più a destra, dieci (10), rappresenta il meglio, il numero più a sinistra, zero (0), rappresenta il peggio. Considerando come sono le relazioni con le persone importanti della tua vita in questo periodo, quale numero sceglieresti?

(peggio)

0

1

2

3

4

5

6

7

8

9

(meglio)

10

**PA:** Considerando come erano le relazioni con le persone importanti della tua vita un anno fa, quale numero sceglieresti?

(peggio)

0

1

2

3

4

5

6

7

8

9

(meglio)

10

**FU:** Considerando come potranno essere le relazioni con le persone importanti della tua vita tra un anno, quale numero sceglieresti?

(peggio)

0

1

2

3

4

5

6

7

8

9

(meglio)

10

**Community Well-Being (CO_WB): satisfaction with one’s community.**

**PR:** Questo gruppo di domande riguarda la tua comunità. Il numero che si trova più a destra, dieci (10), rappresenta il meglio, il numero più a sinistra, zero (0), rappresenta il peggio. Considerando com'è la comunità dove vivi in questo periodo, quale numero sceglieresti?

(peggio)

0

1

2

3

4

5

6

7

8

9

(meglio)

10

**PA:** Considerando come era un anno fa la comunità dove vivi, quale numero sceglieresti?

(peggio)

0

1

2

3

4

5

6

7

8

9

(meglio)

10

**FU:** Considerando come potrà essere tra un anno la comunità dove vivi, quale numero sceglieresti?

(peggio)

0

1

2

3

4

5

6

7

8

9

(meglio)

10

**Occupational Well-Being (OC_WB): satisfaction with one’s job, vocation, or avocation.**

**PR:** Questo gruppo di domande riguarda la tua occupazione principale. Il numero che si trova più a destra, dieci (10), rappresenta il meglio, il numero più a sinistra, zero (0), rappresenta il peggio. Considerando com'è la tua occupazione principale (impiegato/a, lavoratore/rice in proprio, volontario/a, studente/ssa, occuparsi della casa) in questo periodo, quale numero sceglieresti?

(peggio)

0

1

2

3

4

5

6

7

8

9

(meglio)

10

**PA:** Considerando come era la tua occupazione principale un anno fa, quale numero sceglieresti?

(peggio)

0

1

2

3

4

5

6

7

8

9

(meglio)

10

**FU:** Considerando come potrà essere la tua occupazione principale tra un anno, quale numero sceglieresti?

(peggio)

0

1

2

3

4

5

6

7

8

9

(meglio)

10

**Physical Well-Being (PH_WB): state of satisfaction with one’s overall health and wellness.**

**PR:** Questo gruppo di domande riguarda la tua salute e benessere fisico. Il numero che si trova più a destra, dieci (10), rappresenta il meglio, il numero più a sinistra, zero (0), rappresenta il peggio. Considerando com'è la tua salute e benessere fisico in questo periodo, quale numero sceglieresti?

(peggio)

0

1

2

3

4

5

6

7

8

9

(meglio)

10

**PA:** Considerando come era la tua salute e benessere fisico un anno fa, quale numero sceglieresti?

(peggio)

0

1

2

3

4

5

6

7

8

9

(meglio)

10

**FU:** Considerando come potrà essere la tua salute e benessere fisico tra un anno, quale numero sceglieresti?

(peggio)

0

1

2

3

4

5

6

7

8

9

(meglio)

10

**Psychological Well-Being (PS_WB): satisfaction with one’s emotional life.**

**PR:** Questo gruppo di domande riguarda il tuo benessere emotivo e psicologico. Il numero che si trova più a destra, dieci (10), rappresenta il meglio, il numero più a sinistra, zero (0), rappresenta il peggio. Considerando com'è il tuo benessere emotivo e psicologico in questo periodo, quale numero sceglieresti?

(peggio)

0

1

2

3

4

5

6

7

8

9

(meglio)

10

**PA:** Considerando com'era il tuo benessere emotivo e psicologico un anno fa, quale numero sceglieresti?

(peggio)

0

1

2

3

4

5

6

7

8

9

(meglio)

10

**FU:** Considerando come potrà essere il tuo benessere emotivo e psicologico tra un anno, quale numero sceglieresti?

(peggio)

0

1

2

3

4

5

6

7

8

9

(meglio)

10

**Economic Well-Being (EC_WB): satisfaction with one’s financial situation.**

**PR:** Questo gruppo di domande riguarda la tua situazione economica. Il numero che si trova più a destra, dieci (10), rappresenta il meglio, il numero più a sinistra, zero (0), rappresenta il peggio. Considerando com'è la tua situazione economica in questo periodo, quale numero sceglieresti?

(peggio)

0

1

2

3

4

5

6

7

8

9

(meglio)

10

**PA:** Considerando com'era la tua situazione economica un anno fa, quale numero sceglieresti?

(peggio)

0

1

2

3

4

5

6

7

8

9

(meglio)

10

**FU:** Considerando come potrà essere la tua situazione economica tra un anno, quale numero sceglieresti?

(peggio)

0

1

2

3

4

5

6

7

8

9

(meglio)

10
