# Supplementary material for: Measuring subjective well-being from a multidimensional and temporal perspective: Italian adaptation of the I COPPE scale
Source: Health Qual Life Outcomes. 2018 May 8;16:88. doi: 10.1186/s12955-018-0916-9 (PMC5941326; doi:10.1186/s12955-018-0916-9)
Supplement: Supplementary file 2 — Manifest variables Correlation Matrix. (DOCX 18 kb) [file 12955_2018_916_MOESM2_ESM.docx]

| **Additional file 2. Manifest variables Correlation Matrix** | **EC_WB** | **Fu** |  |  |  |  |  |  |  |  |  |  |  |  |  |  |  |  |  |  |  |  | 1 | 7.08 | 2.03 | N.B. All values are statistically significant at the .1% alpha level. Congeneric Variables in boldface. PR = Present; PA = Past; FU = Future. |
| --- | --- | --- | --- | --- | --- | --- | --- | --- | --- | --- | --- | --- | --- | --- | --- | --- | --- | --- | --- | --- | --- | --- | --- | --- | --- | --- |
|  |  | **Pa** |  |  |  |  |  |  |  |  |  |  |  |  |  |  |  |  |  |  |  | 1 | **.468** | 5.92 | 2.05 |  |
|  |  | **Pr** |  |  |  |  |  |  |  |  |  |  |  |  |  |  |  |  |  |  | 1 | **.639** | **.607** | 5.904 | 2.16 |  |
|  | **PS_WB** | **Fu** |  |  |  |  |  |  |  |  |  |  |  |  |  |  |  |  |  | 1 | .281 | .231 | .477 | 7.663 | 1.75 |  |
|  |  | **Pa** |  |  |  |  |  |  |  |  |  |  |  |  |  |  |  |  | 1 | **.325** | .270 | .306 | .205 | 6.069 | 2.08 |  |
|  |  | **Pr** |  |  |  |  |  |  |  |  |  |  |  |  |  |  |  | 1 | **.347** | **.585** | .327 | .221 | .280 | 6.141 | 2.14 |  |
|  | **PH_WB** | **Fu** |  |  |  |  |  |  |  |  |  |  |  |  |  |  | 1 | .323 | .233 | .553 | .267 | .235 | .443 | 7.813 | 1.69 |  |
|  |  | **Pa** |  |  |  |  |  |  |  |  |  |  |  |  |  | 1 | **.436** | .238 | .485 | .257 | .223 | .263 | .234 | 6.732 | 1.84 |  |
|  |  | **Pr** |  |  |  |  |  |  |  |  |  |  |  |  | 1 | **.487** | **.655** | .445 | .252 | .344 | .312 | .269 | .299 | 6.790 | 1.85 |  |
|  | **OC_WB** | **Fu** |  |  |  |  |  |  |  |  |  |  |  | 1 | .260 | .178 | .426 | .336 | .206 | .519 | .343 | .279 | .545 | 7.690 | 1.90 |  |
|  |  | **Pa** |  |  |  |  |  |  |  |  |  |  | 1 | **.392** | .213 | .252 | .184 | .236 | .420 | .218 | .297 | .424 | .216 | 6.229 | 2.13 |  |
|  |  | **Pr** |  |  |  |  |  |  |  |  |  | 1 | **.507** | **.582** | .288 | .177 | .234 | .440 | .248 | .318 | .468 | .324 | .307 | 6.436 | 2.17 |  |
|  | **CO_WB** | **Fu** |  |  |  |  |  |  |  |  | 1 | .264 | .216 | .403 | .217 | .151 | .321 | .270 | .162 | .366 | .250 | .209 | .382 | 6.743 | 2.04 |  |
|  |  | **Pa** |  |  |  |  |  |  |  | 1 | **.601** | .271 | .314 | .246 | .231 | .226 | .232 | .243 | .293 | .206 | .249 | .246 | .221 | 6.074 | 1.81 |  |
|  |  | **Pr** |  |  |  |  |  |  | 1 | **.691** | **.743** | .325 | .255 | .304 | .260 | .175 | .247 | .336 | .190 | .272 | .285 | .236 | .266 | 6.148 | 1.86 |  |
|  | **IN_WB** | **Fu** |  |  |  |  |  | 1 | .248 | .202 | .295 | .206 | .174 | .376 | .307 | .239 | .429 | .363 | .230 | .519 | .205 | .188 | .335 | 8.216 | 1.59 |  |
|  |  | **Pa** |  |  |  |  | 1 | **.477** | .209 | .259 | .187 | .191 | .245 | .165 | .213 | .310 | .244 | .248 | .408 | .244 | .196 | .206 | .168 | 7.220 | 1.74 |  |
|  |  | **Pr** |  |  |  | 1 | **.543** | **.727** | .246 | .197 | .205 | .221 | .195 | .249 | .312 | .230 | .298 | .455 | .234 | .361 | .197 | .175 | .211 | 7.521 | 1.76 |  |
|  | **OV_WB** | **Fu** |  |  | 1 | .327 | .19 | .504 | .300 | .239 | .413 | .339 | .228 | .603 | .292 | .202 | .481 | .407 | .202 | .605 | .315 | .222 | .496 | 7.771 | 1.70 |  |
|  |  | **Pa** |  | 1 | **.274** | .239 | .415 | .201 | .208 | .304 | .173 | .246 | .429 | .216 | .241 | .359 | .211 | .257 | .583 | .242 | .238 | .291 | .177 | 6.306 | 1.86 |  |
|  |  | **Pr** | 1 | **.384** | **.564** | .439 | .255 | .400 | .373 | .270 | .297 | .500 | .283 | .392 | .394 | .245 | .332 | .675 | .290 | .483 | .378 | .287 | .303 | 6.64 | 1.68 |  |
|  | **Variable** | | **Pr** | **Pa** | **Fu** | **Pr** | **Pa** | **Fu** | **Pr** | **Pa** | **Fu** | **Pr** | **Pa** | **Fu** | **Pr** | **Pa** | **Fu** | **Pr** | **Pa** | **Fu** | **Pr** | **Pa** | **Fu** |  |  |  |
|  |  |  | **OV**  **WB** | | | **IN**  **WB** | | | **CO**  **WB** | | | **OC**  **WB** | | | **PH**  **WB** | | | **PS**  **WB** | | | **EC**  **WB** | | | **M** | **SD** |  |
